# Quantifying the Predictive Role of Temperament Dimensions and Attachment Styles on the Five Factor Model of Personality

**DOI:** 10.3390/bs10100145

**Published:** 2020-09-24

**Authors:** Efrat Barel, Yonathan Mizrachi, Maayan Nachmani

**Affiliations:** 1 Department of Behavioral Sciences, The Max Stern Academic College of Emek Yezreel, Emek Yezreel 19300, Israel; 2 Departments of Sociology and Anthropology, The Max Stern Academic College of Emek Yezreel, Emek Yezreel 19300, Israel & The Laboratory for AI, Machine Learning, Business & Data Analytics (LAMBDA), Tel-Aviv University, Tel Aviv 6997801, Israel; yoni1961@post.harvard.edu; 3 Department of Psychology, The Max Stern Academic College of Emek Yezreel, Emek Yezreel 19300, Israel; maayann@yvc.ac.il

**Keywords:** temperament, attachment, personality

## Abstract

Background: The present study investigated the role of temperament and attachment security in predicting individual differences in the five factor personality traits among adults. As previous studies suggested the potential moderating role of attachment in the association between temperament and personality traits, the present study sought to examine an interactionist model combining attachment and temperament in explaining individual differences in personality traits. Methods: A sample of 1871 participants (1151 women and 719 men) completed self-report measures of adult attachment style (the Relationships Questionnaire—RQ), temperament dimension (the Fisher Temperament Inventory—FTI), and personality domain (the Five Factor Model—FFM). Results: Partial correlational analyses revealed associations between attachment security and each of the five domains of the FFM, and few associations between some temperament dimensions and several domains of the FFM. Moderated regression analyses showed that attachment security moderated the associations between temperament dimensions and the Agreeableness domain of the FFM. Among secure individuals, those with higher scores on the Curious/Energetic, Cautious/Social Norm Compliant and Prosocial/Empathetic scales exhibited higher Agreeableness scores, whereas among insecure individuals, those with higher scores on the Analytic/Tough-minded scale exhibited lower scores on the Agreeableness scale. Conclusion: Overall, the current study provides evidence in support of the substantive role of social-environmental factors (Adult Attachment) as a moderating element bridging temperament-related personality elements and a number of their FFM manifestations.

## 1. Introduction

### 1.1. Background

There is a considerable body of literature investigating the determinants of personality traits in adults, which include both environmental and genetic factors [1,2]. A better understanding of the role of parenting behavior, as well as biological factors, in predicting individual differences in typical personality traits has far-reaching consequences for the development of personality disorders [3,4].

The Five Factor Model (FFM) [5] of personality has been consistently favored by personality psychologists over the past decades [6] and has been acknowledged as a useful measure for structuring individual differences in personality [7,8]. The FFM yields five dimensions: Neuroticism, Extraversion, Openness, Agreeableness, and Conscientiousness. Each dimension describes a broad factor of psychological functioning that is composed of a set of more specific facets or traits [9].

Examination of the etiological factors involved in the development of individual differences in personality highlights the importance of heritability [10], as well as early childhood experiences [3]. Developmental theorists postulate that temperament and attachment styles play a key role in explaining individual differences in personality from an early stage of life [11]. Temperament is defined as a heritable pattern of cognitive, emotional, and behavioral dispositions. While research indicates that temperament is influenced by experience [12,13], studies also show that temperament remains largely stable from childhood to adulthood [8,14]. Given that temperament is biologically-based, many psychologists have searched for its physiological foundations [15,16,17,18]. As part of this effort, Fisher and colleagues have recently suggested a novel temperament model, the Fisher Temperament Inventory (FTI), which includes four temperament dimensions: (1) Curious/Energetic, (2) Cautious/Social Norm Compliant, (3) Analytical/Tough-minded, and (4) Prosocial/Empathetic. Each of these dimensions is associated primarily with one of four chemical systems of the brain: (1) dopamine, (2) serotonin, (3) testosterone, and (4) estrogen/oxytocin, respectively [19,20,21]. Brown and colleagues [19] demonstrated the association between the four temperament dimensions of the FTI and the activation of certain brain systems in the predicted brain regions. They found that scores on the Curious/Energetic dimension were associated with activation in a region of the substantia nigra, which reflects activity in the dopamine system. Scores on the Cautious/Social Norm Compliant dimension were associated with activation in the ventrolateral prefrontal cortex, specifically in regions linked with the serotonin system. Scores on the Analytical/Tough-minded scale were associated with activity in regions of the occipital and parietal cortices, which are associated with visual acuity and mathematical thinking, abilities that are linked to testosterone. Finally, scores on the Prosocial/Empathetic scale were associated with activity in regions of the inferior frontal gyrus, anterior insula and fusiform gyrus. These are regions associated with empathy, a trait linked to the estrogen/oxytocin system.

Temperament has been regarded as a direct precursor of personality [22], with some studies suggesting that inheritance accounts for half of the variation in personality dimensions [3]. Developmental studies using prospective approaches to investigate the relationship between child temperament and the FFM have demonstrated that temperament in infancy predicted personality traits in adolescence. For example, infants who showed high sociability were high in emotional stability and openness in adolescence, whereas those who showed resistance to control were less agreeable and more open as adolescents. Additionally, a difficult temperament in infancy was associated with low extraversion in adolescence [23]. Personality studies conducted in adulthood provide further support for the links between temperament dispositions and the FFM [8]. 

Recently, Fisher and colleagues [24] investigated the relationship between the FTI and the FFM. Analyses that were conducted with 215 participants showed that the Curious/Energetic scale of the FTI was positively correlated with Openness and Extraversion, and negatively correlated with Neuroticism. The Cautious/Norm Compliant scale was positively correlated with Conscientiousness and Neuroticism, and negatively correlated with Openness. The Analytic/Tough-minded scale was negatively correlated with Agreeableness and Neuroticism, and positively associated with Openness and Conscientiousness. Finally, the Prosocial/Empathetic scale was positively correlated with Neuroticism and Openness, and negatively correlated with Conscientiousness. 

Apart from the substantial role that temperament plays in the etiology of individual differences in the FFM, parental behavior, and especially the role of attachment to parental figures, has been suggested to be an important factor as well. Attachment theory emphasizes that early experiences with caregivers are crucial to the development of internal working models (IWMs), models that influence how individuals relate to significant others and cultivate interpersonal interactions throughout the life course [25]. Developmental studies on the link between attachment styles in infancy and children’s emerging personalities have shown that attachment security predicted certain dimensions of personality. For example, Hagekull and Bohlin [26] reported that infants’ attachment security (as measured by the Strange Situation Procedure at 15 months) predicted Extraversion, Neuroticism, and Openness when they were children (as measured by mothers’ and teachers’ ratings of the children’s personalities at 8–9 years old). The authors suggested that a secure IWM enables the activation of the exploratory system and, subsequently, the enjoyment that accompanies that exploration [26]; therefore, behaviors that are indicative of extraversion can be seen as expressions of a secure IWM. Furthermore, a secure IWM fosters emotional stability (the opposite pole of Neuroticism), as well as creative and curious behaviors (which correspond to Openness). Adult personality studies using self-report measures provide additional support for these findings [3,27].

Empirical studies have demonstrated the link between temperament and attachment to personality traits. However, very few studies have tested both the biological and environmental precursors of the FFM personality traits in one model. Among the few attempts was Hagekull and Bohlin’s [26] study, in which they conducted a longitudinal study examining the role of attachment and temperament in infancy as predictors of mothers’ and teachers’ personality ratings in childhood. They found that temperament and attachment both predicted individual differences in personality traits, and that the proportion of variance explained by each predictor was relatively similar. Additionally, personality studies with adult participants have investigated temperament and attachment as predictors of personality traits. For example, Picardi and colleagues [27] investigated the role of temperament in predicting attachment and the personality traits of the FFM among 222 adults. They demonstrated that attachment-related anxiety was correlated both with the personality traits of Extraversion and emotional stability, as well as with the temperament dimensions of harm avoidance, reward dependence, and low novelty seeking. Further, Richter, Eisemann, and Richter [28] investigated a model with parental rearing and temperament as predictors of personality characteristics among 540 adults. A factor analysis confirmed the discriminant validity of parental rearing and personality characteristics as different factors. In addition, they found that there were more correlations between parental rearing and personality characteristics, as compared to temperament dimensions. Recently, Haselbeck and colleagues [29] examined the moderating role of attachment style in the association between prenatal maternal stress and child temperament. The results showed that a secure attachment style served as a protective factor and attenuated the effects of prenatal maternal stress on difficult temperament.

### 1.2. The Present Study

The aim of the present study was to investigate the role of temperament and attachment security in predicting individual differences in personality traits. Furthermore, given previous findings suggesting the potential moderating role of attachment in the association between temperament and personality traits, the present study additionally examined the moderating effect of attachment.

## 2. Method

### 2.1. Participants 

In the current study, there were 1871 participants (mean age 29.32 ± 9.98), of which 1151 were women (61.6%) and 719 were men (38.5%). The majority of participants (*n* = 1233) were undergraduate students in the Max Stern Academic College in the northern part of Israel. The rest of the participants were junior and high school students (*n* = 106) as well as individuals aged 30 and above (*n* = 532). The study was approved by the institutional review boards (IRBs) of the Max Stern Academic College of Emek Yezreel (approval number: EMEK YVC 2019-18). “All procedures performed in the study involving human participants were in accordance with the ethical standards of the institutional and/or national research committee and with the 1964 Helsinki declaration and its later amendments or comparable ethical standards”. Informed consent was obtained from all individual participants included in the study.

### 2.2. Instruments and Procedure

Four online questionnaires were used in the study to capture basic demographic information as well as the three distinct personality dimensions. 

Demographic questionnaire. Basic demographic information was collected regarding age and gender, as well as contact information for later follow-up and email addresses to signify participants’ consent to take part in the study.

The Ten Item Personality Measure (TIPI) is a 10-item measure of the Big Five (or Five-Factor Model, FFM) personality traits [30]. It measures five personality factors: Neuroticism (N), Extraversion (E), Openness (O), Agreeableness (A), and Conscientiousness (C), with two items representing each factor. Participants were asked to rate statements on a Likert-type five-point scale, ranging from strongly disagree (1) to strongly agree (5). Sample items included: “I see myself as someone who tends to be lazy” (C) or “I see myself as someone who gets nervous easily” (N). The mean of the two responses for each factor was calculated to produce a numerical score for each personality trait. According to some researchers [31], using 10-item measures are sometimes preferable to using the traditional and longer Big Five measures. The internal reliability (Cronbach’s alpha) for each factor was: 0.42 for Neuroticism, 0.50 for Extraversion, 0.41 for Openness, 0.37 for Agreeableness, and 0.47 for Conscientiousness. The TIPI showed low-to-moderate Cronbach’s alpha, a typical finding in short scales [32]. However, factorial analysis confirmed the five-factor structure underlying TIPI [33,34], and strong correlations with longer personality trait measures were obtained [35]. The TIPI questionnaire was selected to assess both the temperament and character dimensions of participants’ personalities using a lexical-based personality mapping framework [5].

Adult attachment style was assessed using the Relationship Questionnaire (RQ) [36]. The RQ extends the original attachment three-category measure [37] by rewording the descriptions of each of the attachment styles and by adding a fourth style—dismissing-avoidant. The RQ is a single-item measure consisting of four short paragraphs, each describing a prototypical attachment pattern as it applies to close relationships in adulthood. Participants were asked to rate their degree of agreement with each prototype on a 7-point scale. For example, an individual might rate him or herself a six on the Secure description, a two on Fearful, a one on Preoccupied and a four on Dismissing. These ratings (or scores) provide a profile of an individual’s attachment feelings and behavior. The highest of the four attachment prototype ratings is then used to classify participants into an attachment category. Additionally, participants were asked to categorically mark which paragraph best described them, without providing a numerical rating.

Fisher’s personality type was assessed using the Fisher Temperament Inventory (FTI) [19]. The 56-item FTI questionnaire assesses the four broad temperament dimensions: Curious/Energetic, Cautious/Social Norm Compliant, Prosocial/Empathic, and Analytical/Tough-minded. Each dimension is associated with one of four chemical systems of the brain, respectively: (1) dopamine and the related norepinephrine system; (2) serotonin; (3) testosterone; and (4) estrogen and oxytocin. Each of the four categories were assessed with 14 items rated on 4-point rating scale: 1 = strongly disagree; 2 = disagree; 3 = agree; 4 = strongly agree. One sample statement was: “I find unpredictable situations exhilarating” [21]. The internal reliability (Cronbach’s alpha) for each dimension was: 0.79 for Curious/Energetic, 0.83 Cautious/Social Norm Compliant, 0.83 for Prosocial/Empathic, and 0.82 for Analytical/Tough-minded. The FTI questionnaire was selected to assess participants’ “temperament-nature” personality dimension using a biological neural systems-based framework [19].

Our study was approved by the Yezreel Valley College ethics committee (approval number: EMEK YVC 2019-18). Study participants were recruited by third-year B.A. students, who participated in a social science research seminar on personality during the years 2015–2018. After providing their written consent to participate in the study, participants completed four online questionnaires covering basic demographics details, as well as three personality questionnaires regarding temperament, character, and lexical personality dimensions as outlined above.

### 2.3. Variable Assessment

Personality domains, measured by the TIPI, served as the dependent variables. The independent variables were: attachment security and temperament. Attachment security was measured by the RQ scales. The highest of the four attachment prototype ratings was then used to classify participants into an attachment category. The temperament dimensions were measured by the FTI scales. 

### 2.4. Statistical Analyses

First, independent samples *t*-tests were conducted to test for sex differences in the dependent variables (i.e., personality domains) Additionally, bivariate correlations were conducted between participant age and the dependent variables. Next, partial correlation analyses (controlling for sex and age) were conducted in order to investigate the association between temperament dimensions and attachment security (dummy coded with “secure attachment” as the reference group) with individual differences in personality domains. Bonferroni corrections for multiple comparisons were used. Finally, we tested for the moderating role of attachment security in the association between temperament dimensions and personality domains using moderated regression analyses. In order to avoid problems of multicollinearity, the predictor variables were centered before calculating the interaction terms [38]. In the first step, age and sex were included. Next, two variables were included in each regression: attachment security and temperament dimensions. Last, the interaction terms were added to the regression model. Hierarchical regression was employed to determine if the addition of the interaction between attachment security and temperament dimensions improved the prediction of personality domains, over and above their separate effects. Significant interactions were probed using the procedures described by Aiken and West [38].

All analyses were performed using SPSS version 25.

## 3. Results

*Sex differences.* We checked for sex differences in personality. Significant sex differences on each personality domain were found (see Table 1), such that women scored higher on each domain as compared to men. Furthermore, we checked for associations between age and personality domains. Bivariate correlations demonstrated significant results (see Table 1). Therefore, sex and age were included as covariates in all further analyses. 

*Partial Correlations*. Table 2 shows the results of the partial correlation analyses conducted between attachment security and the Big Five personality traits, as well as the four temperament dimensions and the Big Five personality traits, controlling for sex.

*Moderated Hierarchical Regression Analyses.* In order to examine the potential moderating role of attachment security in the association between temperament dimensions and personality domains, a hierarchical regression was conducted. Sex and age were entered in the first step of the model, followed by each temperament dimension and the measure of attachment security in the second step. Finally, the third step included all interaction terms (see Table 3). 

A multiple regression model with Extraversion as the dependent variable, and with the inclusion of the four interactions between attachment security and each of the temperament dimensions, revealed that the addition of the interaction terms did not account for a significant amount of additional variance in the Extraversion scale scores.

A significant interaction was revealed in a multiple regression analysis with Agreeableness as the dependent variable and with the inclusion of an interaction term between attachment security and the Curious/Energetic scale. Simple slope analyses [39] revealed that the Curious/Energetic scale was positively associated with Agreeableness for secure individuals (*b* = 0.01, *t* = 3.08, *p* = 0.002), however, the association was not significant for insecure individuals (*b* = −0.01, *t* = 1.77, *p* = 0.076).

A multiple regression analysis with Agreeableness as the dependent variable and with the inclusion of the interaction between attachment security and the Cautious/Social Norm Compliant scale interaction, revealed a significant interaction. Simple slope analyses revealed that scores on the Cautious/Social Norm Compliant scale were positively associated with Agreeableness for secure individuals (*b* = 0.01, *t* = 3.22, *p* = 0.001); however, the association was not significant for insecure individuals (*b* = −0.00, *t* = 0.07, *p* = 0.947). Therefore, a significant relationship between scores on the Cautious/Social Norm Compliant scale and Agreeableness was found only among secure individuals: higher Cautious/Social Norm Compliant scores were associated with higher scores in Agreeableness.

A significant interaction was revealed in a multiple regression analysis with Agreeableness as the dependent variable and with the inclusion of an interaction term between attachment security and the Analytic/Tough-minded scale. Simple slope analyses revealed that the Analytic/Tough-minded scale was negatively associated with Agreeableness for insecure individuals (*b* = −0.01, *t* = 3.16, *p* = 0.002), however, the association was not significant for secure individuals (*b* = −0.01, *t* = 1.77, *p* = 0.076).

Finally, a significant interaction was revealed in a multiple regression analysis with Agreeableness as the dependent variable and with the inclusion of an interaction term between attachment security and the Prosocial/Empathetic scale. Simple slope analyses revealed that the Prosocial/Empathetic scale was positively associated with Agreeableness for secure individuals (*b* = 0.00, *t* = 3.04, *p* = 0.000), however, the association was not significant for insecure individuals (*b* = −0.00, *t* = 0.74, *p* = 0.460).

A multiple regression model with Conscientiousness as the dependent variable and with the inclusion of interactions between attachment security and each of the temperament dimensions, revealed that the addition of the interaction terms did not account for a significant amount of additional variance in Conscientiousness.

A multiple regression model with Neuroticism as the dependent variable and with the inclusion of interactions between attachment security and each of the temperament dimensions, revealed that the addition of the interaction terms did not account for a significant amount of additional variance in Neuroticism.

A multiple regression model with Openness as the dependent variable and with the inclusion of interactions between attachment security and each of the temperament dimensions, revealed that the addition of the interaction terms did not account for a significant amount of additional variance in Openness.

## 4. Discussion

Previous studies have demonstrated the role of parental behavior, as well as biological factors, in predicting individual differences in personality traits. The present study provided support based on a very large sample for these findings by showing that attachment security and temperament dimensions were associated with personality traits. Specifically, the current study found that attachment security accounted for individual differences across all personality domains of the FFM. Attachment security was positively associated with Extraversion, Agreeableness, Conscientiousness and Openness, and negatively associated with Neuroticism. These results agree with previous studies showing that secure attachment was positively associated with extraversion, agreeableness, and negatively associated with neuroticism [40]. These findings fit well with past observations that based on the perspectives of both child and adult attachment, secure attachment is a crucial precondition for self-directed exploration [41]. Furthermore, DeYoung, Peterson, and Higgins [42] proposed that the Big Five traits are defined by two higher-order meta-traits labeled as stability and plasticity. Stability reflects one’s motivation and ability to maintain stable relationships, and it is marked by higher emotional stability (i.e., lower Neuroticism), Agreeableness, and Conscientiousness scores. On the other hand, plasticity reflects one’s degree of flexibility in behavior and cognition, and it is marked by higher scores on the Extraversion and Openness scales. Recently, Young, Simpson, Griskevicius, Huelsnitz, and Fleck [43] conducted a longitudinal study in which participants’ early attachment styles were assessed using the Strange Situation procedure at 12 and 18 months, and their personalities were later assessed with the Big Five at age 32. Participants who were categorized as having a secure attachment in infancy scored higher on Agreeableness and Conscientiousness, and lower on Neuroticism—traits that reflect the meta-trait of stability—at 32 years old in comparison with participants categorized as having insecure attachment in infancy. The present study provided partial support for these findings. The current findings show that attachment security is associated with the Big Five traits, suggesting its ability to predict individual differences in personality traits. Moreover, the present findings suggest the important role of attachment security in predicting individual differences in the meta-traits of stability and plasticity. Thus far, the literature in the field provides similar results and conclusions.

The literature on temperament characterizes it as a stable trait, representing particular dispositions that influence behavior throughout the life span [44]. Furthermore, temperament has been regarded as a direct precursor of personality [22]. The present findings showed that the Curious/Energetic scale was positively correlated with scores on the Extraversion, Conscientiousness, and Openness scales. Additionally, the Cautious/Social Norm Compliant scale was positively correlated with Conscientiousness and negatively with Openness, and the Prosocial/Empathic scale was positively associated with Neuroticism and Openness. There were no significant correlations between the Analytic/Tough-minded scale and each of the FFM scales. These results partially support previous findings, which use the same inventories to assess temperament and personality traits among adults [24]. The authors suggested that the association between the Curious/Energetic scale and Extraversion scale may be explained by the energetic and risk-taking qualities that are consistent with dopamine system activity, which characterize both curious/energetic individuals [45] and extraverts [46]. Furthermore, based on previous findings presenting the association between Openness scores with the structure and function of specific brain areas that predict working memory performance and attentional control [47], and other findings showing an association between Openness scores and intelligence [48], Fisher and colleagues [24] suggested that these scales share intellectual characteristics which underlie the association between them. With regard to the association between the Cautious/Social Norm Compliant scale and the Conscientiousness scale, the authors suggested that they both evaluate self-control and self-regulation [5], and the need to plan and organize [45]. Other studies using different inventories assessing temperament in infancy [26] and in adulthood [8] have yielded varied results. Nevertheless, they emphasized the role of temperament in infancy in predicting adult temperament and personality [49], and theorized about the corresponding brain infrastructure that underlies both temperament and personality traits [8].

In light of the inconsistencies that characterize the literature on the role of temperament and attachment in predicting individual differences in personality traits, the present study further examined the potential moderating role of attachment in the association between temperament and personality traits through an interactive model. We found that attachment security moderated the association between temperament dimensions and the Agreeableness domain of the FFM. Among secure individuals, those with higher scores on the Curious/Energetic, Cautious/Social Norm Compliant and Prosocial/Empathetic scales also exhibited higher Agreeableness scores. Furthermore, attachment security moderated the association between the Analytic/Tough-minded scale and the Agreeableness domain of the FFM. Among insecure individuals, those with higher scores on the Analytic/Tough-minded scale exhibited lower scores on the Agreeableness scale. The moderating role of attachment security has been previously suggested in developmental studies. For example, Lickenbrock and colleagues [50] have shown that toddlers high in negative reactivity benefitted from having secure attachment. The authors asserted that their results are in line with the differential susceptibility model [51], which suggests that vulnerable children, temperamentally or genetically, would benefit from supportive environments.

### Limitations and Future Directions

The present study has some limitations. First, we found low-to-moderate Cronbach’s alphas for the TIPI scales. This is a common finding when using short measures [32]. However, previous studied examining the psychometric properties of the TIPI reported satisfying temporal stability and high convergence validity with Big Five Inventory [32,52]). Second, the generalizability of the results to the general population is hindered by the reliance upon a sample of predominantly undergraduate students from Israel. Third, our study relied on self-report measures that may be subject to social desirability biases. A prospectively designed study, including objective measures of attachment and temperament, with more demographic and ethnic diverse samples, would help address these limitations. Furthermore, a growing body of research suggests the mediating role of personality traits in the association between parenting behavior and biological factors with later personality disorders. Future studies should include non-clinical as well as clinical participants to enhance generalizability of the findings.

## 5. Conclusions

To summarize, the present data are consistent with previous findings, especially developmental studies, suggesting an interplay between biological factors and parental behavior in predicting personality traits. The implications of the present study relate to both normative and psychopathological development. The etiology of psychiatric disorders includes temperament, attachment, and personality [41], among other factors (e.g., biological). Further research should investigate a broader model that includes the moderating role of social factors in the association between biological factors and personality traits which, in turn, may mediate the development of personality pathology. In order to address this future direction, longitudinal studies are needed to deepen our understanding of the antecedent factors that may influence psychological consequences throughout the life span.

## Figures and Tables

**Table 1:** Means (SD), *t*, correlations and *p* values for sex, age, and personality traits.

	Men (*n* = 719)	Women (*n* = 1151)	*t*	Age (*r*)
Extraversion	3.92 (0.89)	4.02 (0.85)	2.41 *	0.05 *
Agreeableness	3.33 (0.88))	3.43 (0.92)	2.20 *	0.13 ***
Conscientiousness	3.82 (0.93)	3.97 (0.88)	3.63 ***	0.16 ***
Neuroticism	2.70 (0.90)	3.02 (0.95)	7.16 ***	−0.08 **
Openness	3.65 (0.91)	3.75 (0.94)	2.44 *	0.02

* *p* < 0.05, ** *p* < 0.01, *** *p* < 0.001.

**Table 2:** Partial Correlations (controlling for sex and age).

	Extraversion	Agreeableness	Consciousness	Neuroticism	Openness
Secure Attachment	0.33 ***	0.31 ***	0.16 ***	−0.16 ***	0.08 **
Curious/Energetic	0.09 ***	0.04	0.08 **	−0.04	0.12 ***
Cautious/Social Norm Compliant	0.00	0.05 *	0.13 ***	0.00	−0.09 ***
Analytic/Tough-Minded	−0.03	−0.03	0.07 **	−0.05 *	−0.01
Prosocial/Empathetic	−0.01	0.03	0.01	0.09 ***	0.11 ***

* *p* < 0.05, ** *p* < 0.01, *** *p* < 0.001. Correlations that remained significant after performing Bonferroni corrections for multiple comparisons are presented in bold.

**Table 3:** Multiple regression models: Interactions between attachment security and temperament dimensions predicting personality traits of the Five Factor Model (FFM) controlling for sex and age.

	*b*	95% CI	Δ *R*^2^
**Extraversion**			
Secure × Curious/Energetic	−0.00	[−0.01, 0.01]	0.00
Secure × Cautious/Social Norm compliant	−0.00	[−0.01, 0.01]	0.00
Secure × Analytic/Tough-minded	−0.00	[−0.00, 0.00]	0.00
Secure × Prosocial/Empathetic	−0.00	[−0.00, 0.00]	0.00
**Agreeableness**			
Secure × Curious/Energetic	−0.01	[−0.02, −0.01]	0.01 **
Secure × Cautious/Social Norm compliant	−0.01	[−0.02, −0.00]	0.00 *
Secure × Analytic/Tough-minded	−0.01	[−0.01, −0.01]	0.03 ***
Secure × Prosocial/Empathetic	−0.00	[−0.01, −0.00]	0.02 ***
**Conscientiousness**			
Secure × Curious/Energetic	−0.01	[−0.02, 0.00]	0.00
Secure × Cautious/Social Norm compliant	−0.00	[−0.01, 0.01]	0.00
Secure × Analytic/Tough-minded	−0.00	[−0.00, 0.00]	0.00
Secure × Prosocial/Empathetic	−0.00	[−0.01, −0.00]	0.01
**Neuroticism**			
Secure × Curious/Energetic	0.00	[−0.01, 0.01]	0.00
Secure × Cautious/Social Norm compliant	0.00	[−0.01, 0.01]	0.00
Secure × Analytic/Tough-minded	−0.00	[−0.01, 0.00]	0.01
Secure × Prosocial/Empathetic	0.00	[0.00, 0.07]	0.00
**Openness**			
Secure × Curious/Energetic	−0.03	[−0.01, 0.01]	0.00
Secure × Cautious/Social Norm compliant	0.00	[−0.01, 0.01]	0.00
Secure × Analytic/Tough-minded	−0.00	[−0.00, 0.00]	0.00
Secure × Prosocial/Empathetic	0.00	[−0.00, −0.00]	0.00

* *p* < 0.05, ** *p* < 0.01, *** *p* < 0.001. *b* indicates unstandardized regression coefficients. β indicates standardized regression coefficients. CI indicates confidence interval (95% confidence intervals of unstandardized regression coefficients).

## Abbreviations

RQ: Relationship Questionnaire; FTI: Fisher Temperament Inventory; FFM: Five Factor Model; TIPI: Ten Item Personality Measure; N: Neuroticism; E: Extraversion; O: Openness; A: Agreeableness; C: conscientiousness.
